# An ethics training specific for European public health

**DOI:** 10.1186/s40985-015-0008-x

**Published:** 2015-08-25

**Authors:** Victoria Camps, Ildefonso Hernández-Aguado, Angel Puyol, Andreu Segura

**Affiliations:** 1 Universitat Autònoma de Barcelona and Victor Grifols y Lucas Foundation, Barcelona, Spain; 2 Miguel Hernandez University, SESPAS, the Spanish Society of Public Health and Health Management and Ciberesp, Alicante, Spain; 3 Department of Philosophy, Universitat Autònoma de Barcelona, Barcelona, Spain; 4 Health Department of the Catalan Government, Pompeu Fabra University and Ethics and Public Health Task Force from SESPAS, Barcelona, Spain; 5 Departament de Sanitat, Catalonia, Spain

**Keywords:** Public health ethics, Curriculum, Europe

## Abstract

Training in public health ethics is not at the core of public health programmes in Europe. The fruitful progress of the United States could stimulate the European schools of public health and other academic institutions to develop specifically European teaching programmes for ethics that embrace both transatlantic innovations and some adaptations based on the evolution of moral values in European societies. This paper reviews the arguments for a European public health ethics curriculum and recommends the main features of such a programme. Europe shares common values and, above all, the three major ethical principles that were socially and politically crystallized by the French Revolution: liberty, equality, and fraternity. Fraternity, otherwise known as solidarity, although rarely mentioned in the literature on ethical issues, is the moral value that best defines the European concept of public health expressed as a common good, mutual aid, and a collective or shared responsibility for health of the population. Specific political motivations were responsible for the origin of European health systems and for current policy proposals led by the European Union, such as Europe’s commitments, at least in theory, to: reduce social inequities in health and to develop the health in all policies approach. These and other initiatives, albeit not exclusively European, have political and legal repercussions that pose unique ethical challenges. Europe combines homogeneity in social determinants of health with heterogeneity in public health approaches and interventions. It is therefore necessary to develop training in ethics and good government for all public health workers in Europe, especially since a large segment of the population’s health depends on actions and decisions adopted by the European Commission and its regulatory agencies as well as for non EU European Region countries. Based on these arguments, the paper concludes with several recommendations for a common nucleus for the ethics curriculum in Europe.

## Introduction

The influence of ethics on public health is by no means a new issue, although it has not generated either an operative deontology or such a specific application as research ethics or clinical ethics. However, the application of ethical considerations in the most healthcare-related clinical fields has led to a development that, in Gostin’s words, is generated by, for, and in public health [1].

This development affects the different areas of action in public health, which particularly include, according to Callahan & Jennings: 1) the protection and promotion of health (including the prevention of disease); 2) etiological and evaluative research (epidemiology and other types); and, 3) unjust and avoidable social inequities [2] that, according to Upshur, [3] are related above all to the professional dimension and its social and political legitimacy. All of this has a marked orientation towards advocacy of the collective dimension of the population’s health based on equity and social justice and whose practical application must be sufficiently critical to take adequate advantage of the strengths of each of its contributions.

There is thus no doubt about the relevance of ethics in public health and therefore the need for the corresponding training, an issue that was addressed in the previous chapter. Another matter is whether there are currently enough experienced teachers (most of whom, like the bibliography, are of North American origin) and whether a specifically European initiative would be useful. We owe some of the most prominent conceptual considerations of ethics applied to public health as an academic discipline and specifically in relation to bioethics to Dawson & Verweij [4]. They are prominent pioneers in public health ethics issues, proposing a form of ethics that transcends individual considerations in order to consider collective interventions that protect and foster a population’s health. It is not, however, a case of tracking the geographic or cultural origins of the foundations of public health ethics, but rather of considering whether it would be useful to design and develop specifically European teaching programmes for ethics and public health and, if so, why.

As most European schools of public health do not include ethics in their training programmes, at least not in any generalized fashion, and as some of the available programmes adopt a perspective that is closer to clinical bioethics than the community approach that typifies public health, [5] now is the right time to design a training programme.

Although it should take into account the positive experience of the model ethics curriculum observed by North American schools, [6] this design should do more than merely copy it. It is necessary to consider the wide variety of subjects included in the ethics programmes of North American schools of public health [7] and then incorporate the innovations and even some adaptations into the evolution of moral values in European societies. Although ethical foundations and perspectives are universal, the dilemmas faced have their local particularities. For example, regulations for the use of safety belts and helmets illustrate the differences between the expectations, preferences, and values of Europeans and North Americans, which also affect vaccination policies and the rights of immigrants, among other issues.

Below, therefore, are arguments that justify the proposal and that, in observance of the suggestions made by Maecklberghe & Schroeder, [8] share the philosophical perspective of ethics and both the academic and professional view of public health.

## Historical and philosophical arguments

Europe shares a rich cultural history, and its most prominent common values include those that typify the core values of public health and, above all, the three major ethical principles that were socially and politically crystallized by the French Revolution: liberty, equality, and fraternity. People’s liberties and rights regarding health matters were finally recognized in the Nuremberg Code of 1947. This code is a set of research ethics principles for human experimentation that resulted as a consequence of the Nuremberg Trials against the doctors involved in the human experiments in concentration camps as part of the Nazi programs of genocide. The Nuremberg Code laid the foundations for present-day bioethics and the defence of the principle of autonomy. Equality is currently included in most European texts that advocate the right to health protection and universal healthcare. And fraternity, otherwise known as solidarity, is the basis for the welfare state policies shared by European countries [9].

Solidarity is the moral value that best defines the European concept of public health and is expressed as a common good, mutual aid, and a collective or shared responsibility for health. Considering that vulnerability is associated with poor health, public health ethics places special emphasis on the needy sectors of society and on reducing social inequalities. These models are the inspiration for the characteristic welfare state model in European countries,which includes a universal public healthcare system, social policies to reduce health inequalities and community prevention programmes.

This understanding of solidarity contrasts with the values that we usually associate with the individualism and personal behaviours that, at least apparently, typify health policy in the United States of America. Many Europeans find it hard to understand how there can be forty-five million people in that country who do not have medical insurance, which is why we applaud *Obamacare* [10]. Meanwhile, in Europe we tend to feel proud of the solidarity at the core of European health policy, which is extended to social security systems to support the unemployed and the elderly [11]. Paradoxically, solidarity is rarely mentioned in the literature on the ethical issues that arise in public health policy and practice as described by Dawson and Jennings, who suggest that taking solidarity seriously will enrich our approaches to public health ethics [12].

## Political motives

Although European health systems, based on solidarity, are currently in crisis due to budget cuts, the ageing of the population, and also a recent tendency for over-individualism (partly caused, paradoxical though it may sound, by the welfare state itself [13]), the achievement of a sustainable welfare state and public health policies is an ethical imperative supported by fundamental rights. We therefore need to rethink the welfare state model that we want, as well as the measures that should be taken to strengthen solidarity between people [14].

European institutions have, however, shown signs of their commitment to solidarity, which is characteristic of the European culture with regard to health matters. Examples of this are the European Council’s insistence in June 2008 on reducing the differences in terms of healthcare and life expectancy between and within member states, [15] the EU’s Health Strategy, [16] which encourages work to continue on reducing health inequalities, the Announcement by the 2008 Commission on the Renewed Social Agenda, [17] which reasserted Europe’s fundamental ethical objectives in relation to opportunities, equal access, and solidarity; and the 2009 Announcement by the Commission of the European Communities, aimed at the European Parliament, the European Council, the European Economic and Social Committee, and the Committee of the Regions, titled *Solidarity in health: reducing health inequalities in the EU* [18]. Non EU countries of the WHO European Region are in transition but are influenced by EU and WHO standards.

Europe’s commitment to reducing inequalities in social factors that influence health was reflected in the report by the WHO Commission on Social Determinants of Health, [19] supported by certain European presidencies and by the WHO Regional Office for Europe itself, although its demand for specific consideration of public health ethics has yet to be sufficiently reflected in political practice [20–22].

Another European initiative was that by the Finnish Government [23] to highlight health as one of the priorities for public policy, thus reinforcing the lead set by the Ottawa Charter [24]. A perspective, albeit not exclusively European, whose greatest political and legal repercussions have occurred in Europe [25, 26]. Ten years ago, it was noted that there was a need to evaluate the impact on health of interventions in sectors such as urban planning, industry and employment, as well as the public relevance that achieving their introduction to European policy could have [27]. This public health focus obligates us to balance healthcare directly against other social values and to evaluate different political options that have heterogeneous social and economic repercussions. These are challenges, such as water fluoridation and the prevention of injuries, that could benefit from analysis using public health ethics approaches.

Other common challenges are those derived from immigration from outside and within the EU, the free movement of people throughout European territory, health tourism in the EU and policies for the prevention of epidemics and possible natural and other catastrophes. Immigration is at the core of relevant ethical issues, since it is central in the rhetoric of the neo-fascist and xenophobic political parties that are far from being residual in Europe. As Lindert el al remind us, public health has to learn the lessons of ethical failures in Europe and the obligation of the public health community to – in the words of McKee - “not remain silent when others seek to divide us from our fellow human beings” and as Lindert states, “humiliate, separate and murder the ‘others’.” [28, 29].

## Health arguments

In Europe, the determinant agents of population health are, despite their differences, fairly homogenous [30]. Its health services also share the purposes of the welfare state, assuring (through different organizations) a public and accessible supply of health services. Neither is the focus of public health very different in the different European countries, thanks to the work of European health agencies. In this regard, it is worth highlighting the alliance established between the primary care and public health mechanisms in countries like the United Kingdom and Spain, where community health is a common objective of both institutions [31]. The Nuffield Council reported some joint work experiences involving ethics scholars and public health professionals [32]. We should also cite work such as that conducted by the ethics and public health work group at the Spanish Society for Public Health and Health Administration (SESPAS) which, in collaboration with the Grífols Foundation, maintains a platform for joint exchange and deliberation that has already produced tangible results [33, 34].

Beyond the European homogeneities in terms of vision and organization, there are situations and conflicts regarding health issues in Europe that will benefit from an ethical approach. Heterogeneities can enrich the focuses of public health ethics and, when they express conflicts, they can provide a stimulus for ethical considerations to help find balanced solutions. Prominent among these is the diversity of vaccination policies in Europe. The European Centre for Disease Prevention and Control (ECDC), the European Union agency for infectious diseases, provides regular information on the highly diverse national vaccination policies and makes policy recommendations. Despite this, in some cases, there is major heterogeneity in health policy, as is the case with the varicella vaccination, for example. Four countries recommend population-based administration during infancy, including Germany and some regions of Italy and Spain, while other countries like Poland, the United Kingdom, France, and Spain only recommend it for susceptible adolescents and risk groups. Finally, Holland, Sweden, and Norway make no recommendations at all [35]. There are many values and interests involved in the conflicts surrounding the adoption of decisions in each country, along with a wide variety of adopted solutions, and these are worth scrutinizing in terms of ethics. Spain and the United Kingdom, for example, have very different policies. The vaccination is barely administered at all in the countries that do not recommend it in infancy, except in Spain, where a *de facto* alliance between the production company and various scientific societies has, through administration in private healthcare, achieved coverage of 40 %, which represents a risk for the non-vaccinated population, who could be infected at older ages when there is a greater risk of complications [36]. In the United Kingdom, cost-effectiveness studies have produced inconclusive results and the regulator recommended such a low price for the vaccine that the company decided not to market it [37]. The same company, when it failed to be included on the Spanish vaccine schedule, sold it on the private market at the highest price in Europe and achieved very high sales by imitating the strategy initiated by companies producing other vaccinations. Meanwhile, in public health, each country decides on its investments in accordance with different values. For example, for some it is enough for a vaccination to be cost-effective for it to be included on schedules, without considering the opportunity cost, i.e. the value that the investment would have in other health and social areas or even the value that it could provide for other public health interventions. It is in this diverse setting that the population and its free mobility comes into play, which sometimes demands the right to vaccination and rejects measures such as those adopted in Spain to withdraw the vaccination from public sale.

The attitude of European populations to regulations that restrict personal freedom, such as the use of safety belts by drivers and passengers or making helmets compulsory for motorcyclists, [38] is one of general acceptance and would seem worthy of a positive evaluation, while for some North American and European populations such initiatives are an example of unacceptable paternalism [39].

The right to healthcare in Europe is another matter. Although European healthcare models have much in common, the different focuses represent different limitations both among European citizens, depending on what countries they live in, and with respect to people from non-EU countries, the situation of undocumented immigrants being particularly noteworthy [40]. The European Directive on the application of patients’ rights in cross-border healthcare revealed the difficulties imposed on Europe due to the diversity of methods for organizing healthcare [41]. This might be why the preamble stated that no provision of this Directive should be interpreted in such a way as to undermine the fundamental ethical choices of Member States. The ways that these choices are reflected in the treatment of undocumented immigrants are currently putting the values on which European states are based and those of international agreements to the test. Beyond legal issues, the fact is that these challenges are unique and there is no historic background of collaboration in terms of healthcare comparable with what is happening in Europe, so an approach from public health ethics could be highly useful and, consequently, should be considered in the training of people who will work in key positions in European health services.

Meanwhile, European society’s response to its healthcare problems pervades, at least rhetorically, its exterior actions [42]. A glance at the situation confirms that the problems and quandaries arising in global healthcare (intellectual property, emergency action as opposed to action on underlying determinants of health; technological focuses as opposed to basic needs; the fight against disease as opposed to public health services; etc.) require an approach that would benefit from the ethical application of public health from a specifically European perspective.

Even the problems with poor government, such as corruption, tend to be shared [43]. European public health has to deal with the influence of lobby groups and corporations on political decision-making that affects the European population’s healthcare, [44] meaning there is a need for coordinated work. It is therefore necessary to develop training in ethics and good government for all public health workers in Europe, especially since a large amount of the population’s health depends on actions and decisions adopted by the European Commission and its regulatory agencies. This should also apply to non EU European Region countries.

## Recommendations

The political and legislative particularities, as well as those in relation to healthcare, that distinguish the European Region’s population from other populations, justify Europe having its own design that spans a common nucleus for European schools as a whole, which will in turn contribute to the current process of political construction of the new Europe. This nucleus should be complemented by tackling dilemmas of an ethical nature that on a local level are generating conflict between individual and community interests in the different geographical and social scenarios of the continent.

The common nucleus serving as a foundation for an ethics curriculum in European schools of public health should meet the following general objectives:

- ▪ Familiarize graduates with the basic concepts of ethics and of political and moral philosophy sustained by public health ethics.
- ▪ Foster sensitivity to ethics and the acquisition of criteria for the application of ethical considerations among public health professionals in such a way that ethical arguments are integrated into the design and practice of all public health interventions.
- ▪ Improve students’ ability to recognize any ethical tensions and conflicts associated with public health interventions.
- ▪ Provide information to develop skills to enable students to apply ethical values to the analysis of dilemmas and conflicts and, if relevant, to making practical decisions.
- ▪ Facilitate public health professionals’ ability to reflect on their own moral convictions in relation to other health agents and affected populations in a way that fosters debate and negotiation.
- ▪ Respect rules and standards of professional conduct that specifically affect research procedures, which include respect for privacy and confidentiality, as well as the declaration of interests that might sometimes be involved in conflicts.

In this regard, it is useful for the minimum competences to be acquired to satisfy the criteria proposed by ASPHER in 2011, which in summarized form, in terms of cognitive and intellectual competencies, would be as follows:

- ▪ Know and understand the most relevant ethics history, theories and concepts for public health, prominent among which are autonomy, paternalism, induced interventions, individual and collective responsibility, respect for dignity and discrepancy and human rights, as well as the most significant aspects of the history of ethics and its applications, not forgetting the cases of misuse of the principles of public health for political purposes and outright mass murder.
- ▪ Know and understand the criteria and international evidence based “best practices” that enable work by professionals to be qualified as good practice in relation to personal information, confidentiality, privacy or conflicts of interests and in general the ethical dimensions of creating strategies and designing and implementing any public health interventions, as well as those that affect the behaviour of professionals when it comes to assuming personal or institutional responsibilities.
- ▪ Know and understand the nature and characteristics of ethics committees in the field of healthcare and the ethical requirements of for funding or publication of any research project in the field of public health.

As for practical competences, these would be, among others, the demonstrated ability to:

- ▪ Identify and recognize the ethical dimensions and aspects of certain public health policies, strategies and interventions.
- ▪ Include the basic principles of ethics in the creation and design of public health strategies, and in non-discriminatory approaches with respect to the target populations and in the management of human resources.
- ▪ Respect and assume ethical principles with regard to data protection and confidentiality in relation to any information obtained when exercising one’s professional duties.
- ▪ Maintain relations with the system of ethical committees in one’s own country in relation to public health research projects.
- ▪ Bear in mind all the characteristics that may influence ethical dilemmas in the field of public health in Europe.

In short, this is a potential initiative for the useful development of ethics in, by and for European public health fields, not only the European Union. Due to the differences among policies in the European states, this diversity needs to build competencies of public health practitioners in order to learn and to understand neighbours’ approaches.
